# Single-layer KAN for deepfake classification: Balancing efficiency and performance in resource constrained environments

**DOI:** 10.1371/journal.pone.0326565

**Published:** 2025-07-09

**Authors:** Nadeem Jabbar, Sohail Masood Bhatti, Muhammad Rashid, Arfan Jaffar, Sheeraz Akram

**Affiliations:** 1 Faculty of Computer Science and Information Technology, The Superior University, Lahore, Pakistan; 2 Intelligent Data Visual Computing Research (IDVCR), Lahore, Pakistan; 3 Department of Computer Science, National University of Technology, Islamabad, Pakistan; 4 Information Systems Department, College of Computer and Information Sciences, Imam Mohammad Ibn Saud Islamic University (IMSIU), Riyadh, Saudi Arabia; Tianjin University, CHINA

## Abstract

Deepfakes, synthetic media created using artificial intelligence, threaten the authenticity of digital content. Traditional detection methods, such as Convolutional Neural Networks (CNNs), require substantial computational resources, rendering them impractical for resource-constrained devices like smartphones and IoT systems. This study evaluates a single-layer Kolmogorov-Arnold Network (KAN) with 200 nodes for efficient deepfake classification. Experimental results show that KAN achieves 95.01% accuracy on the FaceForensics++ dataset and 88.32% on the Celeb-DF dataset, while requiring only 52.4 MB of memory, 13.11 million parameters, and 26.21 million FLOPs, significantly less than state-of-the-art CNNs. These verified metrics highlight KAN’s potential for real-time deepfake detection on edge devices. Untested capabilities, such as robustness against adversarial attacks, are proposed for future research. This work aligns with the United Nations Sustainable Development Goals, specifically SDG 9: Industry, Innovation, and Infrastructure and SDG 16: Peace, Justice, and Strong Institutions.

## Introduction

### Background

**Fig 1:**
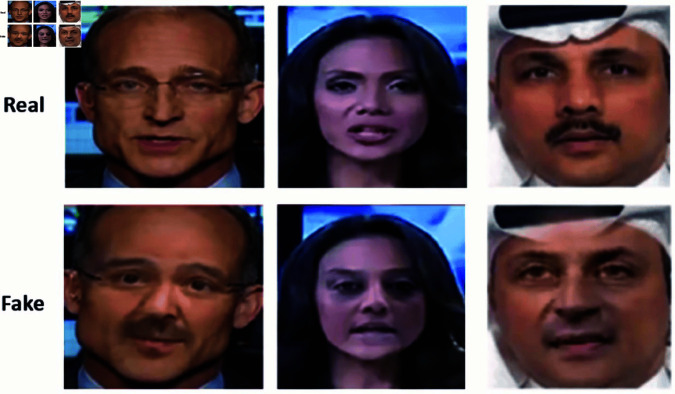
Deepfake generated images [11]. (Images used in this figure are sourced from the FaceForensics++ dataset © 2018, under the MIT License. https://github.com/ondyari/FaceForensics/tree/master).

Deepfake technology generates synthetic images and videos that appear almost indistinguishable from real ones as Fig 1, relies heavily on deep learning methods like Generative Adversarial Networks (GANs). [1] GANs comprise a generator that creates fake media and a discriminator that evaluates its authenticity. Advanced models, such as StyleGAN, have enhanced the visual quality of deepfakes and made detection increasingly challenging over the time [2].

Deepfakes offer potential benefits in entertainment, education, and content creation [3–7], but their misuse poses significant risks including cyberstalking, non-consensual pornography, political manipulation, and misinformation [8]. These threats damage public trust in media and institutions, necessitating robust detection methods, particularly for resource-constrained devices like smartphones and IoT systems. Traditional detection approaches, primarily using Convolutional Neural Networks (CNNs), identify subtle inconsistencies invisible to the human eye [9]. However, CNNs require substantial computational resources, limiting their practicality for edge devices [10]. The rapid evolution of deepfake techniques further complicates detection, requiring models that are accurate and computationally efficient to generalize across diverse datasets and manipulation types.

This study introduces a single-layer Kolmogorov-Arnold Network (KAN) to address these challenges, offering a lightweight, efficient solution for deepfake detection. By achieving high accuracy with minimal resources, KAN aligns with United Nations Sustainable Development Goals. Specifically SDGs 9: which aims to build resilient infrastructure, promote inclusive sustainable industrialization, foster innovation, and SDGs 16: which aims to promote peaceful and inclusive societies.

### Problem statement

Existing deepfake detection methods, predominantly relying on computationally intensive algorithms, are ill-suited for resource-constrained devices like smartphones, tablets, and IoT systems. These devices, ubiquitous in modern society, lack the processing power to support complex models like CNNs. Moreover, detection systems face challenges, including the growing sophistication of deepfake generation techniques, poor generalization across datasets, and susceptibility to adversarial attacks. Continuous research is essential to develop lightweight, accurate, and robust methods that ensure the integrity of digital media and protect against misuse of deepfake.

### Current challenges and shortcomings

Deepfake detection has predominantly relied on Convolutional Neural Networks (CNNs), which excel at extracting spatial features from images. However, CNN-based methods face significant limitations that hinder their deployment on resource-constrained devices and their effectiveness against evolving threats. These challenges include:

#### Computational complexity and resource constraints.

CNNs require substantial computational resources, with millions of parameters demanding high memory and processing power [12,13]. For instance, VGGNet uses 548 MB of memory and 138 million parameters Table 1, rendering it impractical for edge devices. This requires lightweight alternatives optimized for low-resource environments [14,15].

**Table 1:** Resource usage comparison established CNN models and proposed KAN model [25].

Model	Memory Used (MB)	Number of Parameters (M)	Compute Time (ms) Tegra K1 CPU	Compute Time (ms) Jetson TX1 GPU	FLOPs (M)	GFLOPs
VGGNet	548	138	7690	149	15,503	1-2
ResNet	103	25.6	2068	97	3,922	1
AlexNet	61	61	618	20	729	1-2
InceptionNet	53	6.8	1064	35	1,606	1.5-2
Proposed KAN	52.4	13.11	262.16	26.22	26.21	0.013

#### Evolving deepfake techniques.

The continuous advancement of deepfake generation, driven by improved GANs like StyleGAN, increases the realism of synthetic media [2] . Detection methods relying on low-level artifacts become less effective as forgeries become more sophisticated [1], challenging existing systems to keep pace.

#### Limited generalization across datasets.

Many detection algorithms struggle to generalize across datasets or manipulation types. Models trained on specific datasets, like FaceForensics++, often perform poorly on others, such as Celeb-DF, due to overfitting or dataset-specific patterns. This limits their reliability in various real-world scenarios [10].

#### Adversarial attacks and robustness.

CNN-based detectors are susceptible to adversarial attacks, in which minor input perturbations lead to misclassifications. This vulnerability highlights the necessity for robust models that can withstand such manipulations, marking a critical focus for future research [16].

### Objective

The goal of this study is to determine the practical application of Kolmogorov-Arnold Networks (KANs) to improve the accuracy and resilience of deepfake identification in resource-constrained devices. The study will also demonstrate how KANs can be utilized to solve the specific challenges that have been encountered with traditional deepfake detection approaches.

1. In terms of computational efficiency and resource useage, KANs beat other CNN-based approaches for deep fake detection. The hypothesis suggests that the tiny size and efficient architecture of KAN models can provide great approximation potential. Furthermore, KANS also addresses resource requirements and deployment challenges, particularly CNN-based approaches on resource-constrained edge nodes, IoT devices, and embedded systems.
2. Examine how the performance characteristics of KANs, such as accuracy and generalization, compare to traditional deep learning models to identify deepfake content. The hypothesis suggests that KANs will outperform existing methods due to their unique functional representation and the ability to detect subtle inconsistencies in synthetic media [17,18].
3. Analyze whether KANs can effectively adapt rapid advancements in deepfake generation technologies while maintaining their detection capabilities as new techniques emerge. The hypothesis is that the flexible and adaptive nature of KANs will allow them to keep pace with the evolving landscape of deepfake generation [19,20].
4. Identify the practical implementation and scalability challenges associated with deploying KANs for deepfake detection in real-world scenarios. The hypothesis is that KANs will strike a balance between detection accuracy and computational efficiency, facilitating effective real-time deepfake identification [21,22]
5. Compare KANs to existing state-of-the-art deepfake detection techniques regarding performance, interpretability, and computational requirements. The hypothesis is that KANs will demonstrate superior performance while maintaining a high level of interpretability and computational efficiency [23,24].

By addressing these, the study will show the efficiency of KAN as a reliable tool for deepfake detection and, therefore, will contribute to the creation of advanced and accurate approaches to combating the misuse of deepfake technology.

### Contributions

In response to the following key contributions, this study will reveal valuable insight about the effectiveness of Kolmogorov-Arnold Networks as a solution of deepfake detection in resource constrained environments. These findings not only support the development of more effective countermeasures against deepfake exploitation but also contribute to ongoing efforts to enhance security.

- Demonstrating KAN’s computational efficiency, the model achieves high accuracy while requiring only 52.4 MB of memory and 26.21 million FLOPs, making it well-suited for edge devices, Table 1.
- The study shows KAN’s classification accuracy, achieving 95.01% on the FaceForensics++ dataset and 88.32% on Celeb-DF while also demonstrating its capacity to generalize across diverse deepfake types, Table 2 . Importantly, its potential robustness deserves further investigation in future work.
- Comparing KANs to traditional lightweights models and highlighting KAN’s performance gains by spline-based functions, Table 3.
- Analyzing scalability and real-time performance, confirming KAN’s suitability for practical deployment on edge devices, Table 1.
- Identifying deployment challenges, such as dataset limitations, to guide future optimizations.
- Benchmarking KAN against state-of-the-art methods, showcasing superior efficiency and competitive accuracy, Fig 9.
- Evaluating KAN’s adaptability to evolving deepfake techniques, ensuring relevance against new forgeries, Fig 8.

**Table 2:** Comparison of CNN models vs KAN accuracy.

Model Name	DataSet Name	Accuracy	Suitability for Edge Devices
VGG-16	Custom DataSet	86% - 2024 [59]	Less Suitable [60]
VGG-16	FaceForensics++	88% - 2023 [61]	Less Suitable [60]
ResNet-152	Celeb-DF	91% - 2023 [61]	Less Suitable [62]
AlexNet + Additional Layers	FaceForensics++	87.49% - 2020 [63]	Suitable [64]
AlexNet + SVM	Real & Fake Face Detection	86.10% - 2021 [65]	Suitable [64]
InceptionNet	Kaggle DataSet	93% - 2023 [66]	Less Suitable [67]
Inception V3	Custom DataSet	87% - 2023 [68]	Less Suitable [69]
Proposed KAN	FaceForensics++, Celeb-DF	95.01%, 88.32%	Highly Suitable

**Table 3:** Comparison of deepfake lightweight models.

Model	Dataset	Accuracy	Memory Usage	Drawbacks	KAN’s Advantage
Chen *et al*. [29]	Celeb-DF	90.56%	42.8K parameters	- Limited to spatial features; lacks temporal analysis. - Vulnerable to adversarial attacks and struggles with generalization. - Performance on unseen manipulations is unclear.	- Adapts dynamically to diverse manipulations with spline-based activations. - Improved generalization and adversarial robustness while remaining lightweight.
Pipin *et al*. [30]	Face Forensics++, Celeb-DF	97.89%	Not explicitly reported	- High computational cost from PRNU preprocessing. - Performance drops on noisy inputs. - Limited scalability for real-time applications.	- Lightweight (52.4 MB) for real-time edge deployment. - Avoids expensive preprocessing, enhancing efficiency.
Liu *et al*. [31]	Celeb-DF	95%	Not explicitly reported	- Fixed architecture limits adaptability to new deepfake techniques. - Lacks robustness against adversarial attacks.	- Requires fewer parameters (13.11M) and less memory (52.4 MB) with comparable accuracy. - Regularization enhances resilience to noise.
MobiDeep [48]	DFDC	93.4%	2 MB	- Focuses only on eye regions, missing broader manipulations. - Performance declines in low-light or low-res videos. - Not tested on major datasets, limiting comparisons.	- Broader applicability to various deepfake types. - Tested on established benchmarks, providing strong generalization. - Efficient on edge devices (52.4 MB, 26.21 million FLOPs) with good robustness.
KAN (Proposed)	Face Forensics++, Celeb-DF	95.01%, 88.32%	52.4 MB	- - -	- Demonstrates strong efficiency and accuracy for real-time deepfake detection, based with validated results on Celeb-DF and FaceForensics++ datasets. - Optimized for edge deployment with low memory usage and computation time.

**Fig 2:**
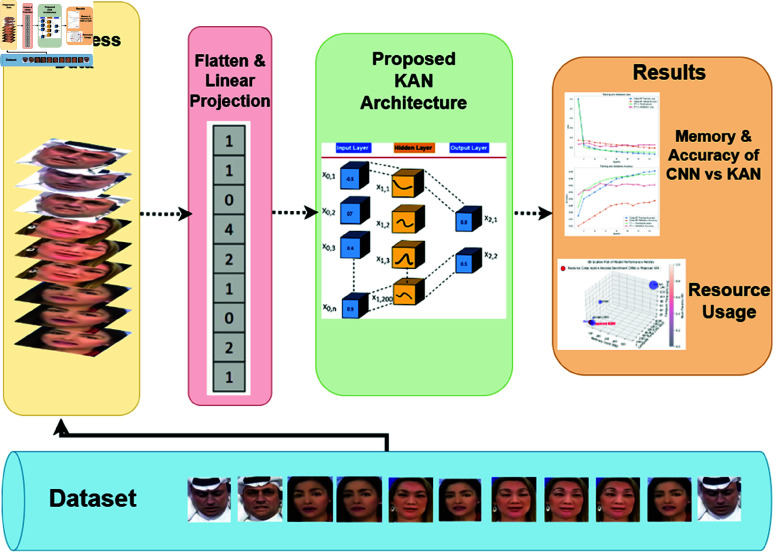
The Comprehensive Overview of the Study’s Methodology (Images used in this figure are sourced from the FaceForensics++ dataset © 2018 , under the MIT License. https://github.com/ondyari/FaceForensics/tree/master)

**Fig 3:**
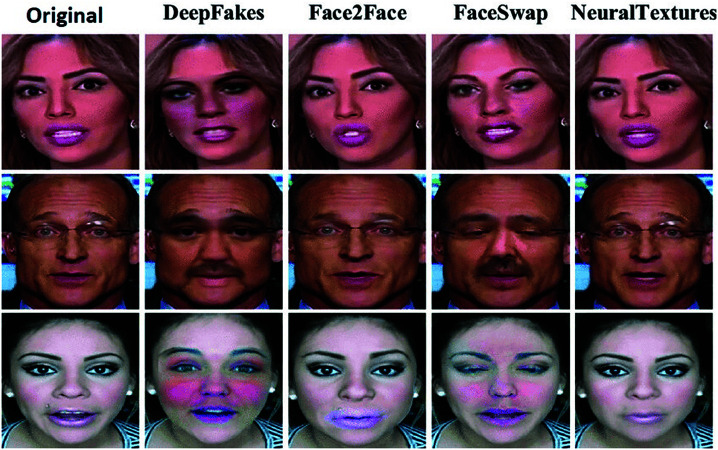
Sample frames of original and four types of manipulations videos from FaceForensics++ (FF++) dataset [11] (Images used in this figure are sourced from the FaceForensics++ dataset © 2018 , under the MIT License. https://github.com/ondyari/FaceForensics/tree/master).

**Fig 4:**
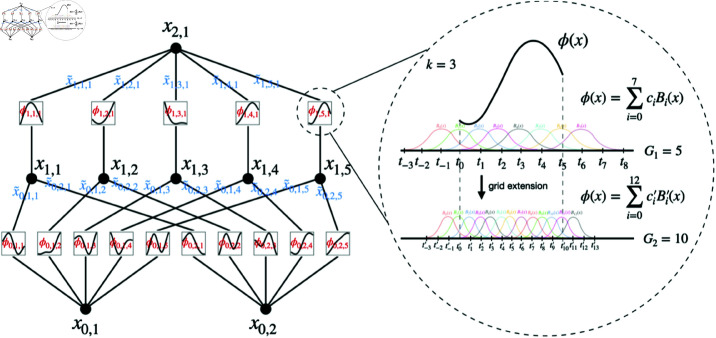
Example of KAN Architecture - Left: Notations for the activations passing through the network. Right: An activation function parameterized with a B-spline [17].

**Fig 5:**
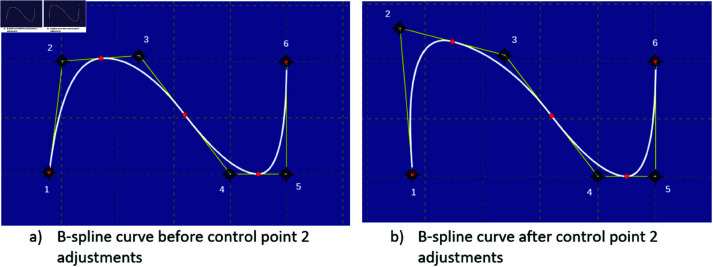
Visual illustration of B-spline curves before and after changing control point 2.

**Fig 6:**
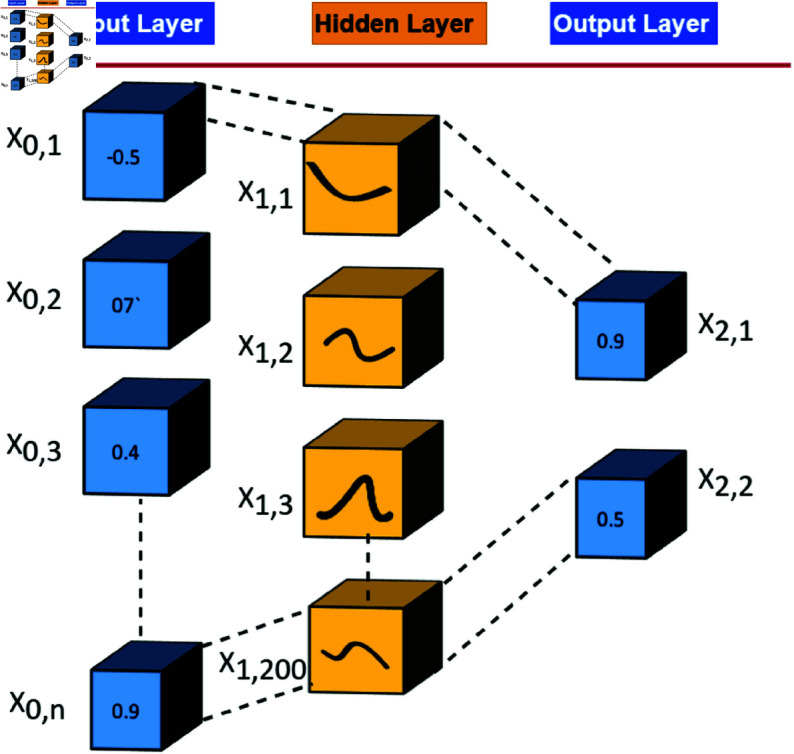
Proposed KAN for deepfake identification on resource constrained environments.

## Related work

### Deepfake detection techniques

Deepfake detection techniques are categorized into visual, audio, multimodal, and emerging techniques. Visual methods analyze inconsistencies in facial movements, blinking patterns, and texture [26]. Audio methods detect anomalies in voice patterns and spectrograms [27]. Multimodal approaches combine both visual and audio data for robust detection [28]. Emerging techniques such as blockchain tracking and adversarial training help ensure authenticity and security of digital content [10].

#### Visual detection techniques.

Recent advancements in deepfake detection using visual techniques have shown promising results across various studies. Chen *et al*. [29] introduced DefakeHop, a lightweight, high-performance deepfake detection method designed specifically for resource-constrained devices. Utilizing the Successive Subspace Learning (SSL) principle, DefakeHop effectively extracted features from facial images with a significantly small model size of 42,845 parameters. The model was evaluated on the UADFV, Celeb-DF v1, and Celeb-DF v2 datasets, achieving an AUC of 100%, 94.95%, and 90.56%, respectively. DefakeHop’s architecture allowed for efficient training and inference, making it highly suitable for mobile and small-device applications, with minimal processing time required for accurate detection. Pipin *et al*. [30] presented a deepfake detection method that leveraged Spatiotemporal Convolutional Networks (SCN) combined with Photo-Response Non-Uniformity (PRNU) analysis. The model was evaluated on FaceForensics++ and the Celeb-DF and DFDC datasets, obtain up to 97.89% accuracy. The lightweight simplicity and efficient processing makes it suitable for integration into small devices. Liu *et al*. [31] introduced a shallow 3D Convolutional Neural Network (3D CNN). Which was proposed for deepfake detection on resource constrained devices. The proposed model was tested on Celeb-DF and FaceForensics++. The major advantage of 3D CNN was the compactness of the architecture, which minimizes storage usage while maintaining high detection accuracy at around 95%. The model’s efficient design making it well-suited for mobile and low-resource devices. Furthermore, Jolly *et al*. [32] develop a CNN-based deep learning model for deepfake detection. The methodology involves training a CNN on a dataset of deepfake and real videos, focusing on identifying visual inconsistencies. The author also combined CNNs with LSTM layers, and Recycle-GAN, achieving over 99% detection rate on FaceForensics++, Face2Face, FaceSwap, and neural texture datasets.In the meanwhile the model faces challenges in detecting highly sophisticated deepfakes. Beside this Ramachandran *et al*. [33] evaluate deepfake detection using deep face recognition techniques. The methodology involves using pre-trained face recognition models to identify inconsistencies in facial features. The study achieved an AUC of 98% on FaceForensics++ and 99% on the Celeb-DF dataset.The study have the limitations include the model’s dependency on high-quality video inputs suggest the future work is to enhance performance on lower-quality videos. Patel *et al*. [34] present a deepfake video detection approach using CNNs and RNNs for temporal irregularities. The authors utilized the Celeb-DF dataset to train and evaluate their deepfake detection model. The results demonstrated the model’s high effectiveness, achieving an accuracy of 98%, precision of 97%, recall of 96%, and an F1 score of 96.5%. The model achieves competitive accuracy but faces challenges in generalizing to unseen deepfake techniques. Jose *et al*. [35] explores deepfake detection using ImageNet models and temporal images of 468 facial landmarks. The methodology involves leveraging pre-trained ImageNet models to analyze temporal sequences of facial landmarks. The limitations include the computational complexity of the used approach.

#### Cutting-edge audio detection techniques.

Recent research has explored various deep learning approaches for detecting audio deepfakes. Bird *et al*. [36] and Lotfi (2023) introduced a novel dataset, DEEP-VOICE, comprising real and AI-generated speech samples. Their real-time deepfake detection system, employing Extreme Gradient Boosting (XGBoost), achieved an impressive 99.3% accuracy in classifying audio samples. Mateen *et al*. [37] proposed a CNN model to classify Urdu audio as genuine or deepfake. using Mel-Frequency Cepstral Coefficients (MFCC) as features and a custom dataset of real and synthetic Urdu audio, they achieved a 91% accuracy in distinguishing between the two. Hamza *et al*. [38] explored the use of MFCC features for deepfake detection, and they tested various machine learning models such as VGG-16. Although the study demonstrated promising results on the fake-or-real dataset, it did not explicitly report accuracy metrics. Chakravarty *et al*. [39] applied a combination of data augmentation approaches and feature fusion to enhanced detection results. They employed MFCCs and PLP feature as an input to the Long Short Term Memory (LSTM) network. The authors attained 99% accuracy with an equivalent error rate of 1.6%. Constant Q Transform (CQT) spectrograms was studied by Abdzadeh *et al*. [40] in the detection of deepfake videos. They audio were then converted to the CQT spectrograms and stored into the Support Vector Machine (SVM). As for the model’s performance, it was tested on the ASVspoof 2019 dataset, and the EER was 3.53%.

#### Multimodal detection techniques.

Multimodal deepfake detection uses multiple types of information, such as video, audio, and text, to improve detection results. Its include video, audio, and text. The suggested method increases the detectors’ dependability by utilizing the complimentary nature of various data to identify fake information. Hyunsoo *et al*. [41] was proposed a method that involved both Vision Transformers (ViT) and CNN through ensemble method. To evaluate the performance of the proposed model, it was trained on DeepFake Detection Challenge (DFDC) dataset and obtained an accuracy of 97.66%. Although the ensemble model had a larger architecture provided by combining two powerful models, it was found to be more generalizable and accurate across different datasets. With parallel computing, the system can be well optimized while maintaining compatibility with small convenient devices. Raza *et al*. [42] proposed a framework named Multimodaltrace in which inputs of audio and visual were processed in the IntrAmodality Mixer Layer (IAML) instruction for multi-label classification. The proposed method was demonstrated to achieve the optimum accuracy of 92.9% on FakeAVCeleb. The cross-dataset accuracy of this model on World Leaders and Presidential Deepfake Detection Datasets yielded accuracies of 83.61% and 70%, respectively. The results also offered an understanding of how the model examined various aspects of audio and visual properties through integrated gradient evaluation. Another deepfake detection method based on multimodal features was proposed by Salvi *et al*. [43] where the authors used both video and audio. The proposed method applies attention-based fusion techniques, where data obtained from the two modalities are fused. Although the approach illustrates the effectiveness of the proposed methods but the article did not describe any datasets, actual figures of performance, and quantitative performance metrics. Muppalla *et al*. [44] explore feature fusion of the audio and the video input to use concatenation and attention mechanisms. However, it also lacks details about the datasets which have been used in this study, and the corresponding performance evaluations have not been elaborated as well. In AV-Lip-Sync+, Shahzad *et al*. [45] used AV-HuBERT to extract features and identify discrepancies in audio and video streams. The method pointed out that lip sync plays a key role in detecting deep fakes; however, this technique also needs further validation on various datasets and comparisons with other state-of-the-art methods. Yang *et al*. [46] proposed AVoiD-DF, a multimodal deepfake detection system that utilizes both audio and visual modalities. The results also highlight the need for feature-level fusion to learn complex interactions between the modalities. The authors employed the DefakeAVMiT, FakeAVCeleb, and DFDC datasets to test the model and reported the state-of-the-art results for deepfake classification.

#### Emerging detection techniques.

Perera *et al*. [47] used super-resolution (SR) techniques to improve image resolution and detect low-quality deepfake movies. The authors utilized a combination of models on the FaceForensics++ dataset, including XceptionNet, VDSR, and RSRGAN. The study confirmed that the combination of RSRGAN with XceptionNet provides a substantial advantage. Improved detection accuracy to 96.05%. The model size was optimized for compact devices, with a focus on SR preprocessing. The model increased its performance against lowquality deepfakes making it suitable for mobile deployment. Mohzary *et al*. [48] developed MobiDeep, a real-time deepfake detection application for portable devices that utilizes corneal-specular backscatter pictures. The model achieved 98.7% accuracy and an average detection time of less than 200ms when solely focusing on the white region of the face, specifically the eyes. The small model size and efficient processing made MobiDeep ideal for mobile deployment, offering robust performance with minimal computational resources. The article [10] tackled the challenge of generalizing deepfake detectors by training them for attribution, which involves distinguishing different types of deepfake attacks. Using datasets such as Google and Jigsaw, FaceForensics++, Celeb-DF, DeepfakeTIMIT, and DF-Mobio, the authors demonstrate that models trained for attribution with triplet-loss generalize better across different databases and generative approaches compared to binary classification models. The results show improved performance on unseen deepfakes, but the method may degrade performance on the same database. Beside it the study [49] addresses generalizable deepfake detection by using self-supervised learning to enrich the diversity of forgeries and improve sensitivity to forgeries. The authors propose adversarial training to dynamically synthesize challenging forgeries, which enhances the detector’s robustness against unseen forgeries. Experiments on various datasets demonstrate superior performance compared to state-of-the-art methods. Limitations include the high computational cost of generating adversarial examples. The publication [26] presents D-VAEGAN, combining variational autoencoder (VAE) and generative adversarial network (GAN) for deepfake detection. The method enhances robustness against adversarial attacks and achieves 96% accuracy on the FaceForensics++ dataset, outperforming existing defense methods. However, further optimization may be required for real-world deployment. Furthermore, The article [50] proposes injecting a universal adversarial signature into generative models to enhance detection and traceability. The method involves adversarial training to embed a unique watermark in generated images. Validated on FFHQ and ImageNet datasets, the method shows promising detection rates. Limitations include the potential impact on image quality. The research [27] investigates techniques for improving the generalization of deepfake detection methods, employing aggressive data augmentation and few-shot tuning. Using datasets from Google and Jigsaw, FaceForensics++, DeeperForensics, Celeb-DF, and DF-Mobio, the authors demonstrate that these techniques improve accuracy in cross-database scenarios. However, the method may require fine-tuning for optimal performance on specific datasets.

### Kolmogorov Arnold network

Liu *et al*. [17] introduce KAN, which represent a significant advancement in neural network architectures, inspired by the Kolmogorov-Arnold representation theorem. KANs replace traditional fixed activation functions with learnable univariate functions parametrized as splines, allowing for more flexible and interpretable models. This innovative approach enables KANs to outperform traditional Multi-Layer Perceptrons (MLPs) in both accuracy and computational efficiency, particularly in data fitting and partial differential equation (PDE) tasks. The comparative analysis presented in [51] highlights that KANs can achieve superior performance with fewer parameters compared to MLPs, emphasizing their practical advantages in high-dimensional data scenarios and complex problem-solving. However, the comparison between KAN and MLP reveals several key differences and similarities. While MLP generally outperforms KAN across various tasks such as machine learning, computer vision, audio processing, and natural language processing, KAN shows a distinct advantage in symbolic formula representation due to its B-spline activation function. This advantage diminishes when B-spline is applied to MLP, enhancing its performance in symbolic formula representation to match or surpass that of KAN. In other tasks where MLP already excels, B-spline does not significantly boost MLP’s performance. Additionally, KAN suffers from a more severe forgetting issue in class-incremental continual learning settings compared to MLP. Both models were compared by controlling the number of parameters and FLOPs, ensuring a fair evaluation. These insights highlight the unique strengths and weaknesses of each model, providing valuable guidance for future research [51]. Recent developments of KANs have received impressive results in the applications to time series prediction and analysis. In [52] the proposed model is tailored for temporal data to take the advantage of KANs architecture and discover temporal dependencies. Subsequent investigation in [23] demonstrates that KAN generated solutions can predict time series data effectively. In [24], KANs has been integrated with a transformer models to yield better results than either of the two alone in terms of accuracy prediction.

Furthermore, a new deep learning algorithm has also been designed for Kolmogorov-Arnold representation. This algorithm presents a step–by–step procedure to decompose multivariable functions into basic forms; this accelerates the advancement of KANs in machine learning theory and application [53]. In the field of graph neural networks and collaborative filtering some new approaches has beed designed by using of KANs. In [54] seeks to enhance the method of feature extraction in graph neural networks by consideration the principles of KAN to learn the structure of the graph. This integration allows for better and more scalable solutions of respective graph-based tasks.

KANs have also been combined with convolutional layers for enhanced image processing capabilities in [55]. Incorporating convolutional operations to these networks, these KANs can preserve the spatial hierarchies and can recognize most complicated structures that exist in the image. which improved the performance of tasks like image classification, object detection and segmentation. CNN–KAN hybrid improves the qualities of the CNN while integrating the facilities of KAN to provide a versatile approach toward image processing applications. Also, Kolmogorov-Arnold Convolutions [56] introduces about the structure and empirical performance of KANs in image associated tasks. Optimised convolutions inside KANs have reported enhanced performance on standard image classification and demonstrating their versatility in solving difficult image challenges.

### Performance tradeoffs between KANs and MLPs

In [17], the authors create a new type of neural network called KAN which is inspired by Kolmogorov-Arnold theorem. KANs deploy learnable spline based activation functions, offering greater flexibility and interpretability compared to traditional multi-layer perceptrons (MLPs). The experimental findings reveal that KANs are superior to MLPs in terms of accuracy and time complexity espacilly in the cases of data fitting and partial differential equations (PDEs).

In support of these results, another benchmarking study made in [18] shows that KANs also work best on tabular data. The study emphasizes their advantages in scenarios requiring interpretability and handling high-dimensional datasets. These studies reveal KANs as a potentially suitable substitute for MLPs in various applications. Runpeng *et al*. [51] critically evaluates the performance differences between KANs and traditional MLPs. The authors argue that previous comparisons may have been biased due to differences in network architecture and parameterization. By standardizing these factors, the study provides a more accurate comparison, highlighting that KANs can outperform MLPs in both accuracy and efficiency, particularly in high-dimensional data scenarios. The findings suggest that KANs are not just theoretically interesting but practically advantageous for various applications.

## Methodology

**Fig 7:**
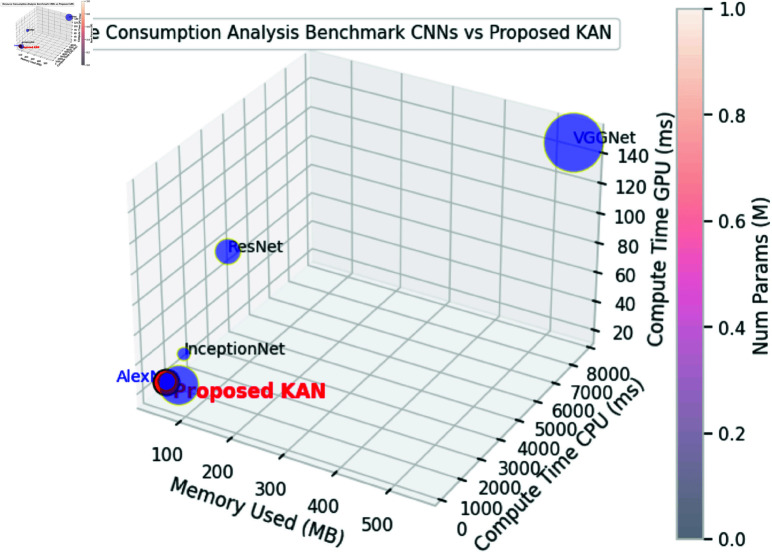
Resource consumption analysis of benchmark CNN models vs proposed KAN.

**Fig 8:**
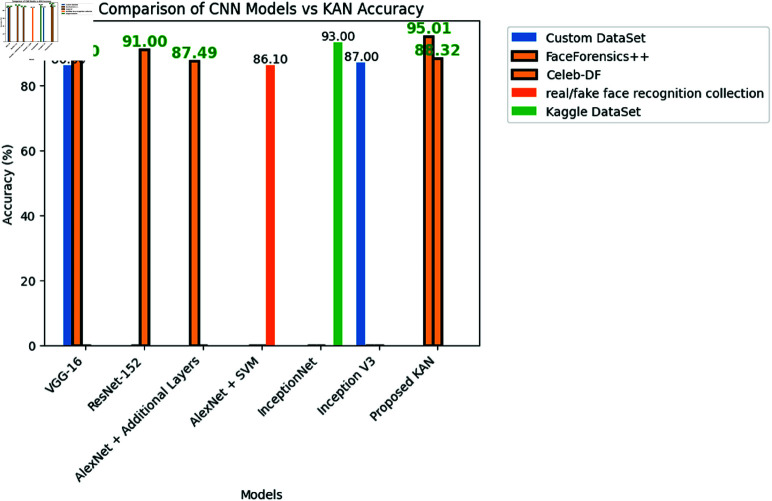
Accuracy of established CNN models vs proposed KAN model.

**Fig 9:**
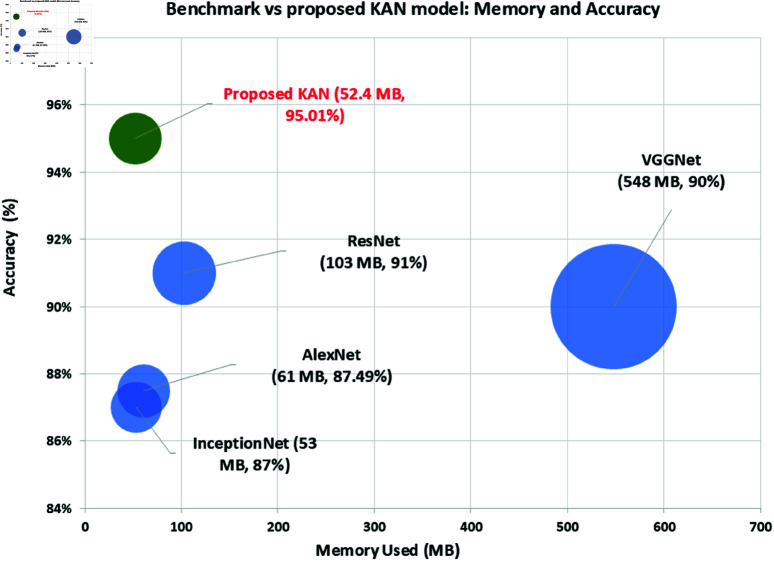
Memory and accuracy of benchmark CNN vs proposed KAN model.

In this study, a comprehensive approach as illustrated in Fig 2 was used to evaluate the performance of the proposed KAN models on two prominent datasets: Celeb-DF and FF++. The results of the study are reflected in Figs 7, 9, 8 and in Tables 2 , 1 to meet the objective as defined in Sect.

### Dataset

1. The FaceForensics++ (FF++) dataset is a large-scale dataset for the evaluation of deepfake detection algorithms [11]. It was collected by the Technical University of Munich and released in 2018. The dataset contains over 1 million video frames.The dataset comprises over 1,000 original videos and four types of manipulation videos: Deepfakes, Face2Face, FaceSwap, and NeuralTextures, with each of 1000. The image of each maipulated type is mentioned in Fig 3. For our study, we extracted individual frames from these videos, resized them to 224x224 pixels, and converted them to grayscale. Data augmentation techniques, such as random cropping, flipping, and rotation, were employed to enhance data diversity. The dataset was divided into training, validation, and test sets using an 80-10-10 ratio. It maintains a balanced distribution of real and fake images, covering a wide range of facial characteristics.
2. The Celeb-DF (Celeb-Deepfake) dataset [57], released by the University of California, Merced in 2019, is a large-scale deepfake video dataset. It is one of the most challenging datasets for deepfake detection. The dataset includes 5639 fake videos and 590 real videos, featuring celebrities with diverse demographics. The deepfake videos were generated using advanced techniques aimed at producing high-quality forgeries. Subjects in the dataset include actors, politicians, and public figures, exhibiting varied facial expressions, head poses, and lighting conditions. To address ethical concerns, the dataset was built using publicly available video data without including any personally identifiable information. The preprocessing includes frame extraction and face alignment from frames, followed by normalization of frames and pixel values and resizing of frames to a standard dimension of 150x150. Low-quality frames and the problem of misaligned faces, which were derived from data quality factors, were respectively solved by filtering and manual correction. The dataset variables include frame number, RGB pixel values, and binary labels indicating the authenticity of each frame. Data splitting was conducted using stratified sampling, with 70% for training, 15% for validation, and 15% for testing.

### Preprocessing steps

This study uses a KAN to detect deepfakes by extracting pixel intensity values at the individual pixel level from grey-scale facial frames of the videos. The data was preprocessed with a high level of preprocessing phases to successfully apply the KAN model provided in this study. Specifically, the details are:

1. The input attributes were standardized to values between -1 and 1. Normalization improves training stability and ensures that all features contribute significantly in the learning algorithm.
2. The control point was calculated using the B-spline interpolation grid as described in Eq 1 and illustrated in Fig 5. This involved selecting the appropriate grid size, range, and additional spaces to accommodate the spline.
3. To improve model stability, the splines’ weights were first set to nonzero using random noise. This method helps to avoid suboptimal solutions.
4. The current base activation function SiLU (Sigmoid Linear Unit), as defined in Eq 2, was applied to them before of linear layers. This activation function facilitates learning by introducing Non-linearity.
5. To limit the risk of overfitting the L1 and entropy-based regularization were implemented.These techniques not only penalize the spline weights but also improve the model’s generalization.

#### Resolution choices.

- The FaceForensics++ dataset is comprises on high quality videos of facial features. Resizing the images from the video’s frames to a size of 224 x 224. These pixels guarantees that enough spatial details are preserved for the purpose of identifying fine details that might have been generated by deepfake techniques like FaceSwap as well as NeuralTextures . This resolution is also complies with the preprocessing standards that are normally used for benchmarking models on this dataset.Furthermore, the said resizing of the data also compatible with existing results in the literature.
- The Celeb-DF videos are more difficult due to quality, lighting and pose may change from one frame to another. Reducing the image to 150×150 minimizes the processing need while keeping those facial features that are required for classification. This choice also solves the problem of the lower-quality frames in the Celeb-DF dataset, where resizing to a higher resolution like 224x224 as FaceForensics++ will amplify noise rather than enhance feature extraction.

### KAN architecture

Kolmogorov- Arnold representation theorem provides the basis on which KAN is constituted, an evaluated model of neural network Fig 4. In contrast to the conventional MLPs that utilize fixed activation functions at their nodes, the KANs employ learnable activation functions on their edges.

The proposed KAN architecture of the study relies on B-spline functions due to its learnable activation functions. Its allow the network to model complex, non-linear relationships in the input data. In the B-spline curve, the control points impart local control over the curve-shape rather than the global control, Fig 5. This spline-based representation is key to the flexibility and efficiency of KAN.

$$spline(x)=\sum_{i}c_{i}B_{i}(x)$$ (1)

The *B*_*i*_(*x*) are the B-spline basis functions, and *c*_*i*_ are the coefficients learned during training. B-splines are piecewise polynomial functions that are defined over a sequence of intervals, called knots. They allow for smooth, continuous representations of input data [17].

$$\text{silu}(x)=\frac{x}{1+e^{-x}}\;$$ (2)

SiLU is used to add a non-linear transformation to the input features before they are processed by the spline function. It helps stabilize learning and introduces flexibility in feature representation.

The Fig 5 presents two B-spline curves: the first in Fig 5a shows the curve prior to any adjustments, while Fig 5b illustrates a new curve resulting from the movement of control point 2. Notably this adjustment maintains the overall shape and continuity of the curve, highlighting a fundamental characteristic of B-splines, local control. Modifying a single control point does not significantly impact the entire curve, demonstrating the stability and robustness of B-splines in shape modeling and KAN’s performance.

$$f(x)=\sum_{q=1}^{2n+1}\Phi_{q}\left(\sum_{p=1}^{n}\phi_{q,p}(x_{p})\right)$$ (3)

This is the core equation of the KAN. Each input feature *x*_*p*_ is transformed by the spline function $\phi_{q,p}(x_{p})$, then aggregated using higher-level transformations $\Phi_{q}$.

ϕ(x)=w·(b(x)+spline(x)) (4)

Here, *b*(*x*) is the base activation function (SiLU), and spline(x) is the spline-based transformation. The weight *w* controls the influence of the spline.

$$b(x)=\text{silu}(x)=\frac{x}{1+e^{-x}}$$ (5)

This reiterates that the SiLU function serves as the foundational non-linearity.

The proposed model was defined by using the KAN as illuetrated in Fig 6. It was designed by PyTorch. The input size was set to 256 x 256. The output layer had two nodes, indicating the model was designed for image classification tasks. The model had a single hidden layer with 200 units, followed by an output layer with 2 units, corresponding to a binary classification task. The AdamW optimizer was used in the study for optimization. The learning rate was set to 1e-3, and the weight decay was 1e-4. In addition, exponential learning rate scheduler to reduce the learning rate during training, using a gamma parameter of 0.8. The standard cross-entropy loss was chosen as the objective function for the binary classification task. The KAN architecture utilized B-spline basis functions and spline-based weights. This provided a powerful and flexible framework for learning and classification tasks.

### Training process

We started by experimenting with different grid sizes, such as 3, 5, and 7. We also apply different spline orders like 2, 3, and 4. Additionally, we adjusted scaling factors for noise, base weights, and spline weights. We tested various activation functions, including ReLU, LeakyReLU, and SiLU. Furthermore, we varied the batch size (e.g., 32, 64, 128), the number of epochs (e.g., 10, 15, 20), and the image size (e.g., 150x150, 224x224, 256,256, 512x512). Extensive experimentation revealed that a grid size of 5, spline order of 3, and SiLU are optimal. It was also determined that the ideal batch size was 64 after setting the number of epochs to 15.These hyperparameters were found to improve the model’s effectiveness and generalization capabilities. Further testing is needed to evaluate their impact on resistance to adversarial attacks. To keep things simple, the model architecture contained only one hidden layer, Fig 6. We tried several values to determine the nodes of hidden layer like: 100, 200, and 300, but 200 seemed to perform the best. Several optimizers were tested on the network, including SGD, Adam, and AdamW. The AdamW optimizer, with a learning rate of $1\times 10^{-3}$ and weight decay of $1\times 10^{-4}$, achieved the best performance. We utilized an exponential learning rate scheduler with a decay rate of 0.8 to adaptively control learning rate during training. To prevent overfitting, regularization techniques were utilized to adjust spline weights during training. These included L1 regularization, which penalized the absolute values of the weights, and entropy-based regularization, which ensured a balanced weight distribution. During training sessions, validation was tracked and the results analyzed in order to improve the training process of the model. Through careful tuning of the hyperparameters and following the training process, as outlined above, the KAN model managed to learn from the input data and reached high accuracy of the predictions while being quite insensitive to the changes in the data and capable of generalizing to unseen examples.

## Resource estimation methods for CNN models

The deploying of CNNs on resource-constrained devices, such as edge devices and embedded platforms, requires a careful estimation of computational resources. Key metrics include memory usage, floating-point operations (FLOPs), power consumption, and energy usage. The following section describes the formulas used to estimate these metrics.

### Memory usage

A significant portion of memory in a CNN is utilized for storing model parameters, such as weights and biases, as well as the feature maps, which are the outputs of each layer. The memory required for parameters could be calculated [25] as:

$$\text{Memory (MB)}=\frac{\text{Number of Parameters}\times \text{Precision (bits)}}{8\times 10^{6}}$$ (6)

where the precision was typically 32 bits for floating-point operations.

### FLOPs (Floating Point Operations)

FLOPs measured the computational cost of CNNs, representing the number of multiply-accumulate (MAC) operations required. For a convolutional layer, the FLOPs could be calculated as:

$$\text{FLOPs}=2\times \text{H}\times \text{W}\times {\text{C}}_{\text{in}}\times {\text{K}}_{h}\times {\text{K}}_{w}\times {\text{C}}_{\text{out}}$$ (7)

where H and W were the dimensions of the output feature map, ${\text{C}}_{\text{in}}$ and ${\text{C}}_{\text{out}}$ were the input and output channels, and ${\text{K}}_{h}$ and ${\text{K}}_{w}$ were the kernel height and width [25,58].

### Compute time

To understand the performance of CNNs on edge devices, we used a tool called Augur. Augur estimates the resource requirements, including compute time and memory usage for any given CNN configuration [25]. It achieves this by analyzing the descriptor of a CNN model and using corresponding profiling data. By simulating various configurations, Augur provides insights into the most efficient ways to deploy CNNs on edge devices. This tool can also determine when performance optimizations or offloading computations are necessary, ensuring efficient execution of deep learning tasks on resource-constrained devices.

### Application to CNN models

These formulas were applied in various studies to model and optimize the deployment of CNNs on mobile and embedded platforms. For example, [25] adopted these equations in their Augur tool to predict the resources needed for execution of ConvNets on portable devices. Likewise, [58] used these formulas to assess the computational costs of various neural network architectures.

Table 1 provides information for the amount of memory used (in MB), the number of parameters in (M), computation time in (ms) on Tegra K1 CPU, computation time in (ms) on Jetson TX1 GPU, number of FLOPs in (M) and GFLOPs for different CNN models and the proposed KAN model at the edge devices.

## Experiments and results

### Experimental setup

The NVIDIA Tegra K1 CPU and Jetson TX1 GPU are important to consider for the utilization of edges devices, particularly relevant to CNN resource requirements. The Tegra K1 CPU is powered by a 3.2GHz quad-core ARM Cortex-A15 32-bit processor and paired with a 192 CUDA cores Kepler GPU along with 2GB of DDR3L RAM. The Jetson TX1 GPU has a more powerful configuration with a 1.9GHz quad-core ARM Cortex-A57 64-bit CPU, 256 CUDA cores Maxwell GPU, and 4GB of LPDDR4 RAM.

These components, commonly found in developer kits like the NVIDIA Jetson Tegra K1 and Jetson TX1 boards. These are necessary for operation and enhance the performance of CNN models such as AlexNet, VGG-Net, GoogleNet and ResNet etc. Both platforms support CUDA with indicating GPU acceleration, which is critical for high-performance deep learning tasks. The Tegra K1 and Jetson TX1 provide for efficient monitoring and evaluation of time, memory, and power consumption for different CNN models. It is beneficial to study these in order to identify many areas that can be improved. This analysis helps to installing CNNs on edge devices, as well as potential solutions for improving system and low-power resources. By understanding these limitations the developers can optimize their CNN models for efficient performance on limited resource platforms.

### Evaluation metrics

#### Evaluation metrics for resource usage.

To evaluate the KAN model’s efficiency with davailable resources, we compare it to baseline dmodels using appropriate metrics. The metrics are included Model Memory Used (MB), Number of Parameters in million (M), Compute Time in millisecond (ms) on Tegra K1 CPU and on Jetson TX1 GPU, FLOPs in Millions (M), and GFLOPs. The results are summarized in Table 1.

- Model Memory Used: We describe memory costs for each model in megabytes (MB) to better understand resource usage during inference on edge devices. Less memory consumption indicates a more efficient model. That is particularly important for deployment on resource-constrained devices.
- Number of Parameters: The number of parameters used in the model is described in millions (M). The size of parameters during training and inference are strongly related with resource constrained environment. The models with fewer parameters are generally more efficient, faster and reducing the computational burden, but accuracy must be maintained.
- Compute Time: Compute time is evaluated on two different hardware platforms: the Tegra K1 CPU and Jetson TX1 GPU, Sect. The execution time, measured in milliseconds (ms). It is crucial for understanding the model’s performance on low-power, CPU-based devices, which are commonly used in edge computing.
- FLOPs: Floating Point Operations (FLOPs) measure in millions (M) for the number of arithmetic operations required to perform inference. Lower FLOPs indicate a more efficient model in terms of computational complexity.
- GFLOPs: Giga Floating-Point Operations Per Second (GFLOPs) metric represents the number of floating-point operations per second in billions. Specifically, 1 GFLOP equals 1 billion FLOPs. The lower GFLOPs indicates that fewer computations are needed, which can be beneficial in resource constraints devices because it reduces power consumption, speeds up processing time, and conserves energy.

#### Evaluation metrics for CNN accuracy.

The effectiveness of the proposed KAN model is evaluated through a comprehensive comparison with several established CNN models. The evaluation is based on the accuracy achieved across different datasets and the published year of each model to provide context for the technological advancements in the field.

- Accuracy: The accuracy is a fundamental metric that measures the proportion of correct predictions made by the model out of the total predictions. Higher accuracy indicates better performance and reliability of the model in correctly classifying or predicting the outcomes. The accuracy of the model was calculated using the following formula:
  $$\text{Accuracy}=\frac{\text{TP}+\text{TN}}{\text{TP}+\text{FP}+\text{TN}+\text{FN}}$$ (8)
  The accuracy of a binary classification model is often calculated using the formula of 8 where: True Positives (TP) represent instances where the model correctly predicted a positive class. True Negatives (TN), on the other hand, indicate instances where the model correctly predicted a negative class. False Positives (FP) occur when the model incorrectly predicts a positive class when it was actually negative. Finally, False Negatives (FN) represent instances where the model incorrectly predicts a negative class when it was actually positive.
- Publish Year: The publishing year of each model provides context for the technological advancements in the field. Furthermore, its also provides context regarding the model’s recency and relevance, as newer models incorporate more advanced techniques and optimizations.

### Results

The proposed KAN model was thoroughly evaluated against of well-established CNN models to demonstrate its significance performance in terms of resource utilization and accuracy across different datasets. The summary in Tables 1 and 2 , serves to highlight the computational efficiency and model’s effectiveness in deepfake detection tasks, respectively. The results are aligned with the paper’s objective of introducing a model that balances accuracy with resource optimization.

#### Resource usage comparison.

The resource usage comparison for different CNN models, including the proposed KAN, reveals its significane into their efficiency and performance, Fig 7 and Table 1. The KAN model demonstrates exceptional memory efficiency, using only 52.4 MB, which is considerably lower than VGGNet (548 MB), ResNet (103 MB), AlexNet (61 MB), and InceptionNet (53 MB). This low memory usage makes the KAN model highly suitable for deployment on resource-constrained devices. Furthermore, the KAN model has only 13.11 million number of parameters, striking a balance between complexity and efficiency. It has fewer parameters than VGGNet (138 million), ResNet (25.6 million) and AlexNet (61 million), but more than InceptionNet (6.8 million).

When evaluating compute time on the Tegra K1 CPU, the KAN model shows the fastest time of 262.16 ms, outperforming VGGNet (7690 ms), ResNet (2068 ms), AlexNet (618 ms), and InceptionNet (1064 ms). This superior performance is also evident on the Jetson TX1 GPU, where the KAN model achieves a compute time of 26.22 ms, significantly faster than VGGNet (149 ms), ResNet (97 ms), and InceptionNet (35 ms) but more than AlexNet (20 ms). The KAN model’s efficiency is further highlighted by its low computational complexity, requiring only 26.21 million FLOPs, compared to VGGNet (15,503 million), ResNet (3,922 million), AlexNet (729 million), and InceptionNet (1,606 million). Additionally, the KAN model achieves 0.013 GFLOPs, underscoring its efficiency in terms of computational resources.

#### Efficiency evaluation and comparison.

**Fig 10:**
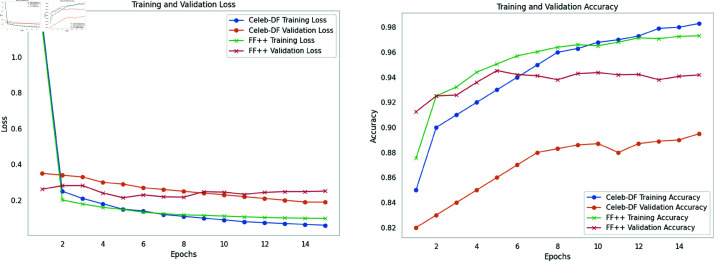
Training and validation metrics for Celeb-DF and FF++ datasets of proposed KAN.

The performance of the proposed KAN was compared to several established CNN models across various datasets, with a focus not only on accuracy but also on resource consumption, demonstrated in Figs 8, 10 and Table 2.

The results reveal that traditional models such as VGG-16, ResNet-152, and InceptionNet deliver strong accuracy in deepfake detection tasks, they do so at the cost of high resource usage as mention in Table 1. For instance, VGG-16 achieved an accuracy of 90% on a custom dataset and 88% on the FaceForensics++ dataset. ResNet-152 reached 91% accuracy on the Celeb-DF dataset, and InceptionNet recorded a high accuracy of 93% on the Kaggle Dataset. However, these models require significant computational resources, making them less practical for deployment in resource constrained devices.

AlexNet, despite enhancements such as additional layers and integration of support vector machines (SVM), achieved a more modest accuracy of 87.49% on the FaceForensics++ and 86.10% on real and fake face detection datasets, respectively. Although AlexNet is less resource intensive than models like VGG-16 and ResNet-152, its accuracy falls short, particularly in complex deepfake detection tasks.

In contrast, the proposed KAN model demonstrates that high accuracy does not necessarily require high resource consumption. KAN achieved an impressive accuracy of 95.01% on the FaceForensics++ dataset and 88.32% on the Celeb-DF dataset. These results are competitive with, and in some cases exceed, the performance of other leading CNN models. More importantly, KAN does so with significantly lower memory usage, fewer parameters, and faster computation times.

As mentioned in Table 3 Chen *et al*. [29] suggest DefakeHop, which utilizes the Celeb-DF dataset, achieves an accuracy of 90.56% with 42.8K parameters. However, it has notable drawbacks, including its limitation to spatial features and a lack of temporal analysis. This model struggles with generalizing to newer deepfake techniques and is vulnerable to adversarial attacks. While it demonstrated good performance on Celeb-DF, it reflects difficulties with unseen manipulations.KAN employs spline-based activations that dynamically adapt to diverse manipulations, which we hypothesize may offer better generalization and potentially improved adversarial robustness compared to some other models. However, specific tests for adversarial robustness are required to confirm it. Pipin *et al*. [30] report an impressive accuracy of 97.89% on the FaceForensics++ and Celeb-DF datasets, but this comes at a high computational cost due to PRNU preprocessing. Additionally, the model requires high-quality inputs and is less robust when dealing with noisy or compressed videos, which limits its scalability for real-time edge applications. KAN stands out with its lightweight architecture, using only 52.4 MB of memory, enabling real-time deployment on edge devices without relying on computationally expensive preprocessing techniques. Liu *et al*. [31] model, also tested on Celeb-DF, achieves an accuracy of 95%. It is however constrained by a fixed framework that cannot be changed. The solution does not address robustness against adversarial attacks or noise in new deepfakes. Though it uses a moderate amount of memory but KAN needs the smallest number of parameters (13.11 M) while obtaining comparable or even better accuracy. Additionally, KAN employs regularization techniques to enhance its robustness against adversarial and noisy inputs. MobiDeep [48], which is trained on the DFDC dataset, likewise achieves 93.4% accuracy. The mobile version of the study is characterized that it is uses less than 2 MB of memory. However, it focuses on eye locations and excludes other manipulations like body gestures. It depends only on the corneal specula’s bright reflection. That will reduces its efficiency in low-light conditions or with occlusions such as spectacles. The lack of testing on popular benchmarks like Celeb-DF and FaceForensics++ make its performance comparisons tough.Furthermore, the model is subject to adversarial attacks on specific aspects, as well as having issues on low-quality and compressed data. In contrast, the proposed KAN model does not depend on domain-specific features, allowing broader applicability to various deepfake types. It has been evaluated against popular benchmarks and demonstrating its capacity of generalization. The study deployment on edge devices results in 52.4 MB of memory with 26.21 million FLOPs. Additionally, it mitigates noise and adversarial attacks to give consistent and reliable solutions. MOreover, the usage of B-splines allows for dynamic feature representation. The suggested KAN model performs well on both FaceForensics++ and Celeb-DF datasets, maintaining the accuracies of 95.01% and 88.32% respectively. Its robustness and precision make it ideal for real-time and edge device operations.

## Discussion

### Interpretation of results

The primary objective of this study was to develop and evaluate the KAN as an efficient model for resource constrained devices and an accurate model for deepfake detection. The experimental results validate the effectiveness of KAN, demonstrating its ability to achieve high accuracy on diverse datasets with minimal computational resources, represented in Figs 7, 9 and Table 1. Specifically, KAN achieved an accuracy of 95.01% on the FaceForensics++ dataset and 88.32% on the Celeb-DF dataset, closely matching or even exceeding the performance of leading CNN models such as ResNet-152 and InceptionNet V3, Figs 8, 10 and Table 2.

Moreover, KAN’s ability to maintain such accuracy while significantly reducing resource consumption, which is presented in Table 1, lower memory usage, fewer parameters, and faster computation times perfectly aligns with the study’s objective. These results highlight KAN’s potential as a practical solution for deepfake detection, particularly in environments where computational efficiency is critical. The insights gained from these experiments confirm that KAN successfully balances the trade-off between high detection accuracy and resource efficiency, making it a valuable contribution to the field of deep learning and digital media forensics. Our findings demonstrate that KANs outperform existing methods in terms of resource efficiency, accuracy, and generalization for classifying various types of deepfake data. However, their robustness against adversarial attacks was not specifically tested and remains an area for future research.

### Challenges and limitations

The current approach presented in this study faces several challenges and limitations, particularly regarding the optimization of the KAN Mapping Layer. Extensive experimentation was required to ensure that the model could generalize effectively when exposed to different data types. This process was crucial in achieving an optimal balance between accuracy and resource efficiency. One of the significant challenges is that deepfake generation is an ongoing process, not a one-time event. As a result, continuous updates in detection processes are necessary to ensure the effectiveness and accuracy of deepfake media classification. This challenge highlights the need for ongoing research focused on developing robust KAN models that can effectively address the dynamic nature of the deepfake landscape. Additionally, another limitation lies in the scope of the datasets used in this research. Although KAN has demonstrated strong generalization and efficiency on the FaceForensics++ and Celeb-DF datasets, its performance on other forms of manipulated media and newer deepfake techniques remains untested. Future studies should explore these areas to further validate the model’s adaptability and robustness in evolving real-world scenarios.

### Future work

Future research should focus on addressing the limitations identified in the current study. For example, refining the KAN Mapping Layer would enhance the model’s ability to capture non-linear data structures. Additionally, extending the research on KAN to incorporate a wider variety of datasets and applying it to different forms of media manipulation and deepfake scenarios will be essential in validating its generalization. Furthermore, focus on designing and implementing tests with various methods of adversarial attack to assess and enhance the model’s resilience, addressing a critical gap in current evaluation. To validate the robustness of KAN, it is crucial to extend the research by incorporating a wider variety of datasets and applying it on different forms of media manipulation and deepfake scenarios. Another promising avenue for development is enhancing the KAN model itself and integrating it with complementary approaches, such as temporal sequence analysis or multi-modal data fusion, to make it less sensitive to new methods of deepfake generation. Moreover, exploring the deployment of KAN in real-time and low-latency applications, especially on edge devices could significantly extend its applicability and impact.

## Conclusion

This study explored the use of KANs as a computationally efficient and robust approach for deepfake detection.Experimental results demonstrate that KAN with a single hidden layer of 200 nodes achieves impressive accuracy of 95.01% on the FaceForensics++ dataset and 88.32% on the Celeb-DF dataset. These results are competitive with and, in some cases, exceed the performance of traditional CNN models. The KAN model also demonstrates significant efficiency advantages, requiring only 52.4 MB of memory, 13.11 million parameters, and 26.21 million FLOPs. In contrast, traditional CNNs typically require substantially higher computational resources, making KANs particularly suitable for resource-constrained environments. Through extensive experimentation on these datasets, KANs demonstrated their ability to match or surpass CNN performance while utilizing significantly fewer resources. The efficiency and accuracy of KANs highlight their potential as a scalable solution for deepfake detection. However, despite these promising results the development process encountered specific obstacles, such as optimizing the Mapping Layer for effective generalization across diverse datasets and the constrained scope of the datasets. Which focused solely on visual deepfakes and excluded audio and multimodal forgeries. Additionally, although the architecture of KANs suggests possible resilience to adversarial attacks, this aspect has not yet been empirically tested. To address these limitations, future research will prioritize several key areas: (1) validating KANs’ robustness against adversarial techniques such as the Fast Gradient Sign Method (FGSM), DeepFool and Projected Gradient Descent (PGD); (2) broadening the dataset to encompass a wider variety of manipulated media, including audio and multimodal deepfakes; and (3) enhancing the model through architectural refinements and the integration of temporal or multimodal analysis to improve adaptability and real-time performance. Furthermore, this study aligns with the United Nations Sustainable Development Goals, particularly SDG 9: Industry, Innovation, and Infrastructure and SDG 16: Peace, Justice, and Strong Institutions, by promoting secure and innovative solutions in digital media forensics.
